# Do Differences in Social Environments Explain Gender Differences in Recreational Walking across Neighbourhoods?

**DOI:** 10.3390/ijerph16111980

**Published:** 2019-06-04

**Authors:** Fatima Ghani, Jerome N Rachele, Venurs HY Loh, Simon Washington, Gavin Turrell

**Affiliations:** 1 International Institute for Global Health, United Nations University, Kuala Lumpur 56000, Malaysia; 2 Centre for Health Equity, School of Population and Global Health, The University of Melbourne, Carlton, VIC 3010, Australia; j.rachele@unimelb.edu.au; 3 Institute for Physical Activity and Nutrition, School of Exercise and Nutrition Sciences, Deakin University, Geelong, VIC 3220, Australia; venurs.loh@deakin.edu.au; 4 School of Civil Engineering, The Faculty of Engineering, Architecture and Information Technology, University of Queensland, Brisbane, QLD 4072, Australia; hos@civil.uq.edu.au; 5 Healthy Liveable Cities Group, Centre for Urban Research, RMIT University Melbourne, Melbourne, VIC 3001, Australia; gavin.turrell@rmit.edu.au

**Keywords:** gender equality, recreational walking, social environment, between-neighbourhood variation, multilevel modelling, random coefficients, urban planning, ecological interventions, sustainable development goals, sustainable cities and communities

## Abstract

Within a city, gender differences in walking for recreation (WfR) vary significantly across neighbourhoods, although the reasons remain unknown. This cross-sectional study investigated the contribution of the social environment (SE) to explaining such variation, using 2009 data from the How Areas in Brisbane Influence healTh and AcTivity (HABITAT) study, including 7866 residents aged 42–67 years within 200 neighbourhoods in Brisbane, Australia (72.6% response rate). The analytical sample comprised 200 neighbourhoods and 6643 participants (mean 33 per neighbourhood, range 8–99, 95% CI 30.6–35.8). Self-reported weekly minutes of WfR were categorised into 0 and 1–840 mins. The SE was conceptualised through neighbourhood-level perceptions of social cohesion, incivilities and safety from crime. Analyses included multilevel binomial logistic regression with gender as main predictor, adjusting for age, socioeconomic position, residential self-selection and neighbourhood disadvantage. On average, women walked more for recreation than men prior to adjustment for covariates. Gender differences in WfR varied significantly across neighbourhoods, and the magnitude of the variation for women was twice that of men. The SE did not explain neighbourhood differences in the gender–WfR relationship, nor the between-neighbourhood variation in WfR for men or women. Neighbourhood-level factors seem to influence the WfR of men and women differently, with women being more sensitive to their environment, although Brisbane’s SE did not seem such a factor.

## 1. Introduction

Gender is a consistent predictor of physical activity (PA) in adults, with women being less active than men across the life-course [1,2,3,4], regardless of whether PA is measured objectively or subjectively [5]. There are biological reasons (in terms of biological structure, function and psychosocial development) for which women are less active than men [6], commonly referred to as differences attributable to sex [7]. However, while gender often refers to the socially constructed norms, roles and relations of men and women, it could also be understood as an attribute impacted by the interaction between biological factors and the wider determinants of health, arising from socioeconomic and environmental structures [8].

Previous research suggests that women experience more individual and environmental barriers to PA [9,10], and the social environment seems to influence their PA more than men [11]. The marked gender disparity in overall PA participation [1,2,4] is acknowledged within the World Health Organization’s (WHO) *Global action plan for the prevention and control of non-communicable diseases 2013*–*2020* [12] and *Women, Ageing and Health: A Framework for Action* [13]. These frameworks call for ecological evidence to inform gender-responsive multilevel strategies (i.e., structural, behavioural or psychological) to increase PA in populations. 

Walking is the most common form of PA among adults [14] and seems to be preferred by women [15]. Walking contributes more towards meeting the current PA guidelines of 150 min or more per week at moderate or brisk pace [16] in women than men [17], whereas men are more likely to participate in vigorous-intensity PA [4]. 

Regular walking contributes to daily energy expenditure [18], reducing or postponing morbidity and mortality from non-communicable diseases [19,20], particularly among women [21]. As walking is typically undertaken within the local neighbourhood [22], environmental features might facilitate or inhibit residents’ walking patterns [23]. 

The factors that influence walking operate at multiple levels and differ depending on whether the intention for walking is recreation or transportation. This paper focuses on walking for recreation (WfR), which is usually undertaken discretionarily in outdoor settings for the purpose of leisure, exercise, or enjoying the scenery [24] and therefore, it is likely to be more influenced by an individual’s perceptions of the neighbourhood’s social context than objectively measured built environmental factors [24,25,26,27]. The social environment comprises residential characteristics related to the social interactions among its residents, which are important in promoting healthy cohesive communities [28]. Social environment features form part of the liveability indicators, which make a community desirable to live in [29]. Liveability indicators, in turn, align with the social determinants of health currently being examined within social–ecological frameworks to inform healthy and equitable urban design and policy [30]. 

Previous multilevel research observed that WfR varies by gender, with women more likely to walk for recreation than men [31,32]. To date, most neighbourhood-based studies have presented the overall (average) association between gender and WfR, overlooking the possibility that this relationship differs depending on the characteristics of neighbourhood environments. However, a previous investigation revealed that the effect of gender on WfR varied significantly across neighbourhoods [32], suggesting that the overall relationship was not necessarily reflective of the association within any particular neighbourhood. Moreover, the overall effect was potentially obfuscating important information about how neighbourhoods differentially influence the WfR of men and women. 

Several studies have explored gender as a moderator in the relationship between the social environment and WfR, with stronger environmental effects observed in women [23,33]. This suggests that more social environment support might be required to encourage women to walk for recreation as a strategy for reducing the gender disparity in overall PA participation. Perhaps favourable social environments for walking generate minimal or no gender differences in WfR, whereas larger gender differences in WfR might be observed in socially fractured environments. Therefore, the impact of the neighbourhood social environment on a person’s probability of WfR might vary by gender. In other words, gender differences in WfR might be moderated by the social environment (one that varies only between neighbourhoods). 

Furthermore, between-neighbourhood variation of gender differences in WfR might be attributed to gender-specific sensitivity to environmental characteristics, reflecting the fact that men and women experience—and engage with—their local environments in distinct ways [34,35]. Thus, it is plausible that the social environment of a neighbourhood might have a stronger influence on the recreational walking of women compared to men. For instance, women typically have more concerns about personal safety [3], especially at night [36], which is likely to influence their recreational walking [37]. By contrast, neighbourhood safety seems to have either no impact [38] or an inverse effect [31,33] on men’s WfR. 

Consistent with the principles of social-ecological models, which posit dynamic interrelations across multiple levels of influence [39], this study investigated the contribution of the social environment to explaining: (1) Neighbourhood differences in the gender–WfR relationship; and (2) between-neighbourhood variation in WfR for men and women. 

## 2. Materials and Methods 

### 2.1. Study Design and Data Collection

This investigation uses data from the second wave (collected in 2009) of the How Areas in Brisbane Influence healTh and AcTivity (HABITAT) longitudinal multilevel study of mid-age adults living in Brisbane (Australia). HABITAT is underpinned by a social–ecological framework, which informs the investigation of the relative contributions of environmental, social, psychological, and sociodemographic factors on PA patterns. Details of HABITAT’s sampling design have been published elsewhere [40]. Briefly, a multistage probability sampling design was used to select a stratified random sample (*n* = 200) of Census Collection Districts (CCDs), or ‘neighbourhoods’, with a random sample of people aged 40–65 years from each CCD subsequently selected (85 persons on average). Eligible participants were mailed a survey between May and July of 2007 using a method developed by Dillman [41]. Of the 16,127 in-scope participants, 11,035 valid responses (68.4%) were received at baseline (collected in 2007), and of the 10,837 in-scope participants in the second wave, 7866 valid responses (72.6%) were received in 2009 (Wave 2). The baseline sample was broadly representative of the Brisbane population [42]. The HABITAT Study received ethical clearance from the Queensland University of Technology Human Research Ethics Committee (Ref. No. 3967H and 1300000161). 

### 2.2. Measures

#### 2.2.1. Outcome Variable

Walking for Recreation (WfR): A single question asked participants to report the total time (converted to minutes) spent walking for recreation, leisure or exercise in the previous week. This question was closely modelled on the one used in the Active Australia Survey, which has demonstrated reliability [43] and validity against accelerometer measures [44], and has been recommended for Australian population-based research [45]. The WfR variable was positively skewed and included outlier values, which were top-coded to 840 minutes as recommended [46], equivalent to a maximum of two hours of daily walking. Only 1.3% of participants in the analytical sample reported WfR for longer than 840 mins per week. Exploratory analysis of WfR revealed two relatively discrete groups as previously used [47]: one reporting 0 mins of WfR in the previous week, and another reporting 1–840 mins.

#### 2.2.2. Independent Variable

Participants were asked to identify as either male or female, which was considered thereafter the gender variable in the analyses.

#### 2.2.3. Measures of the Neighbourhood Social Environment

The social environment was conceptualised through measures of individual perceptions of social cohesion, incivilities, and safety from crime aggregated to the neighbourhood (or CCD) level. These area-level exposures are the most commonly used social environment factors in neighbourhood-based research assessing WfR [1,48,49,50]

Perception of social cohesion: participants were asked to respond to eight Likert-type items, ranging from strongly disagree to strongly agree, which measured perceptions of neighbourliness, trust in neighbours, shared values, and friendships and relationships with neighbours. *The following statements are about your suburb and the people living around you. How much do you agree or disagree with each statement? I have a lot in common with many people in my suburb; If I no longer lived here, hardly anyone around here would notice; I am good friends with many people in my suburb; I generally trust my neighbours to look out for my property; I have little to do with most people in my suburb; Most of the time, people in my suburb try to be helpful; Generally speaking, people in my suburb can be trusted; Most of the time, people in my suburb just look out for themselves.*

These items closely reflect those in the Buckner Social Cohesion Scale [51], with acceptable reliability [52]. The items were subjected to a Principal Components Analysis (PCA) with Varimax rotation and combined to form a weighted linear scale (Cronbach’s alpha of 0.85).

Perception of incivilities: this was assessed through two Likert-type items that asked participants about the presence of litter or rubbish, and graffiti in the neighbourhood. *The following statements are about your suburb’s surroundings. How much do you agree or disagree with each statement? Please tick the box that best applies to your suburb: My suburb is generally free from litter or rubbish: My suburb is generally free from graffiti.* The items have acceptable reliability [53] and the resultant PCA scale had a Cronbach’s alpha of 0.63.

Perceptions of safety from crime: using the aforementioned approach, participants responded to six Likert-type items that asked about their neighbourhood’s level of crime, whether it was a safe place for adults to walk during the day and at night, and if children were safe. *The following statements are about crime and safety in your suburb. How much do you agree or disagree with each statement? There is a lot of crime in my suburb; Children are safe walking around the suburb during the day; The crime in my suburb makes it unsafe to walk streets at night; Rowdy youth on streets or hanging around in parks in my suburb; The crime in my suburb makes it unsafe to walk streets during day time; I would feel safe walking home from bus stop/train station at night.* The items were adapted for the Australian population from the Neighbourhood Environment Walkability Scale [54], which has acceptable reliability [53,55]. The PCA scale created from these items had a Cronbach’s alpha of 0.80.

Several of the items comprising the social environment scales required reverse coding to follow the same direction, which was undertaken prior to the creation of the social environment scales. For analytic purposes, as the focus of this study was on whether different social environments (i.e., an ecological exposure) influence gender differences in WfR across neighbourhoods, neighbourhood-level perceptions of social cohesion, incivilities, and safety from crime were derived using a mean scaled score for each of the 200 neighbourhoods. An Empirical Bayes Exchangeable (EBE) estimation method was applied. This method, described in detail in previous studies [56,57], produces more precise estimates of the neighbourhood social environment than a simple aggregated mean score. It is based on the independence assumption where random effects are regarded as exchangeable (i.e., assumes neighbourhoods to be exchangeable in borrowing strength). This estimation method is superior to a mean aggregated score previously used in studies of the social environment [58,59,60,61] (such as the Ordinary Least Squares estimator, which relies solely on the information from each neighbourhood in estimating that neighbourhood’s latent variable [62]), as it produces a mean neighbourhood social environment score that not only accounts for the number of participants per neighbourhood, but also the variability of the exposure within and between neighbourhoods [62]. Spatial dependence was not considered due to the sparsity of neighbourhoods included in the study throughout the Brisbane area. 

Calculation of the EBE estimate involved four steps [62] reported in an earlier HABITAT study [56] as follows:

1. Creating a mean score of the exposure for each neighbourhood (${\bar{Y}}_{.j}$);
2. Using an ANOVA model of the exposure, fitted using maximum likelihood to obtain estimates of the between –and within– neighbourhood variance. This was then used to obtain an estimate of the reliability of the exposure estimate ${\hat{\lambda }}_{Ej}$ for each neighbourhood, using Equation (1), where ${\hat{\tau }}_{E}$ is the between-neighbourhood variance, ${\hat{\sigma }}_{e}^{2}$ the within-neighbourhood variance, and $n_{j}$ the number of informants within the neighbourhood;
3. Estimating the exposure intercept ${\hat{\gamma }}_{E}$; and
4. Calculating the EBE estimate using Equation (2)

Equation (1):

(1) $${\hat{\lambda }}_{Ej}=\frac{{\hat{\tau }}_{E}}{\left({\hat{\tau }}_{E}+\frac{{\hat{\sigma }}_{e}^{2}}{n_{j}}\right)}$$

Equation (2):

(2) $${\hat{\beta }}_{EBEj}={\hat{\gamma }}_{E}+{\hat{\lambda }}_{Ej}\left({\bar{Y}}_{.j}-{\hat{\gamma }}_{E}\right)$$

The social environment measures were correlated within the 200 HABITAT neighbourhoods (Pearson correlation *r* = 0.44–0.76, *p* < 0.001); thus, each measure was modelled separately.

Furthermore, a previously used approach considering social environment exposures as continuous measures in the statistical analyses [63] was replicated to ensure comparability between studies as recommended [50]. Therefore, the average effects can be interpreted as the likelihood of WfR for every 1 standard deviation (SD) unit increase in social environment.

**Figure 1:**
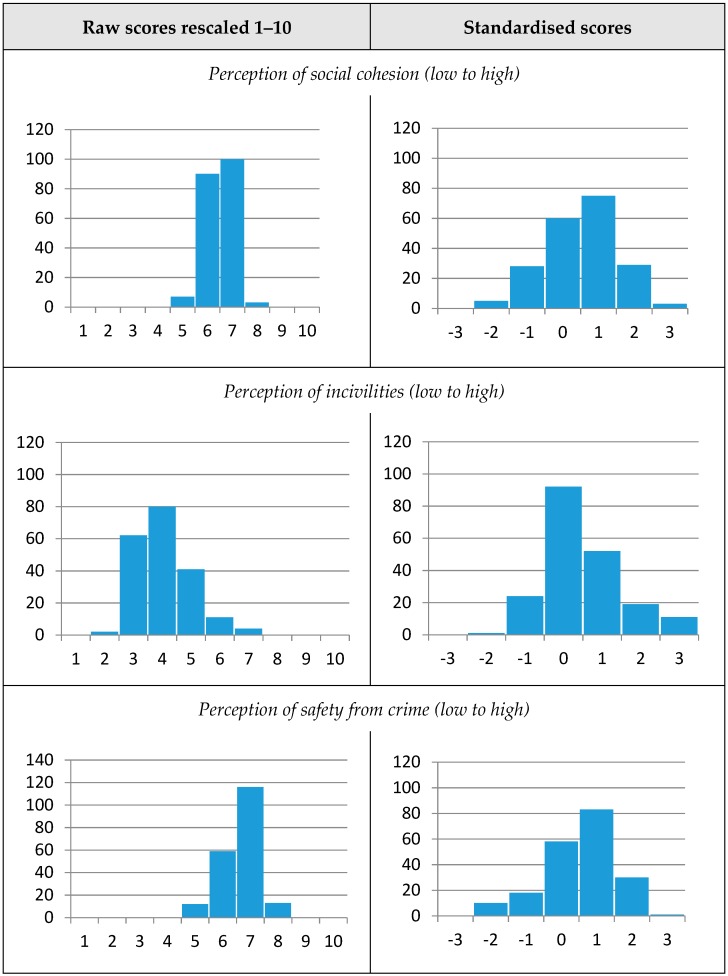
Distribution of social environment exposures (x axis) across the 200 HABITAT neighbourhoods (y axis).

The social environment scales were operationalised in two ways. First, for descriptive purposes, the raw social environment scores of the 200 neighbourhoods were re-scaled to range from 0–10, where 10 represents the highest score on each scale (Figure 1). The raw scores for social cohesion across the 200 neighbourhoods ranged from 4.5 to 7.1 (mean 6.0, SD 0.5) with a high score representing high perception of neighbourhood social cohesion; while for incivilities it ranged from 1.5 to 6.4, (mean 3.5, SD 0.9) with a high score representing high perception of neighbourhood incivilities; and for safety from crime it ranged from 4.4 to 7.7, (mean 6.2, SD 0.6) with a high score representing high perception of neighbourhood safety from crime. Second, the three scales were standardised for comparison, and revealed that the social environment was distributed over a relatively narrow range, with most of the neighbourhoods located within 1 standard deviation from the mean, indicating limited variation. 

#### 2.2.4. Covariates

Participants reported their date of birth from which a year of age in 2009 was derived. The age for the analytical sample ranged from 42 to 67 years, with a mean of 53.7 years (SD 7.0).

Education: respondents provided the highest educational qualification attained, which was coded as follows: (1) Bachelor’s degree or higher (including postgraduate diploma, master’s degree, or doctorate), (2) Diploma (associate or undergraduate), (3) Vocational (trade or business certificate or apprenticeship), and (4) No post-school qualifications.

Occupation: respondents provided their job title, which was classified according to the Australian and New Zealand Standard Classification of Occupations (ANZSCO) [64] and recoded into five categories: (1) Managers/professionals (managers and administrators, professionals and paraprofessionals); (2) White-collar employees (clerks, salespersons and personal service workers); (3) Blue-collar employees (tradespersons, plant and machine operators and drivers and other labourers and related workers); (4) Not in the workforce (home duties and retired); and (5) not easily classifiable (not employed, students, permanently unable to work or other category). 

Household income: respondents provided an estimate of the total pre-tax annual household income through a question comprising 13 income categories. For analysis, these were re-coded into the following six categories: (1) ≥AU$130,000, (2) AU$129,999–72,800; (3) AU$72,799–52,000; (4) AU$51,999–26,000; (5) ≤AU$25,999; and (6) not classified (including blank responses, ‘Don’t know’ or ‘Don’t want to answer’).

Residential self-selection: to assess residential attitudes, participants were asked to respond to five Likert-type items at baseline (data collected in 2007), ranging from ‘not at all important’ to ‘very important’ on a number of statements regarding “How important were the following reasons for choosing your current address?”. PCA with Varimax rotation identified three factors whose items had loadings of 0.50 or above, as recommended [65], and these were subsequently described as ‘destinations’ (three items referring to ease of walking to places, and closeness of public transportation and shops, Cronbach’s alpha = 0.80) ‘nature’ (three items, Cronbach’s alpha = 0.78) and ‘family’ (two items, Cronbach’s alpha = 0.61).

Neighbourhood-level disadvantage: each of the 200 neighbourhoods was assigned a socioeconomic score using the Australian Bureau of Statistics’ Index of Relative Socioeconomic Disadvantage (IRSD) [66]. The Index reflects each area’s overall level of disadvantage based on 17 socioeconomic attributes, including education, occupation, income, unemployment, and household tenure. The derived socioeconomic scores from the HABITAT neighbourhoods were then quantised as percentiles relative to all of Brisbane [40] ranging from 1–100 (with a mean of 56.8 and SD 28.0), with lower scores denoting more disadvantaged neighbourhoods. 

### 2.3. Statistical Analyses

Of the 7866 participants who returned a valid questionnaire in 2009, the following were excluded from the analyses: 568 (7.2%) relocated from their original neighbourhood at baseline (2007) to another address in 2009; and 162 (2.2%) were identified as being a different participant from who responded at baseline, with most of these participants also having incomplete data on education and age. Of the remaining 7136 eligible participants, several had incomplete data on education (*n* = 19), on WfR (*n* = 199), and on residential self-selection variables (*n* = 275), giving a total of 493 missing records (6.9% of the eligible participants). Sensitivity analyses revealed that participants who were not classified for occupation (*p* = 0.001) or income (*p* = 0.012) were significantly more likely to be in the missing group of 493. 

A listwise deletion (rather than multiple imputation) was applied to the 493 missing records based on the following rationale: the missing data approached the recommended 5% threshold for imputation [67]; the original sample was broadly representative of the target population [42]; the efficiency gains offered by applying missing data methods (which add another layer of measurement error to the data) are often minor in large samples [68]; and the analytic sample remained large enough to address the study aims.

The final analytical sample comprised 6643 participants nested within 200 neighbourhoods, and the demographic characteristics are presented in Table 1. The number of respondents per neighbourhood ranged from 8 to 99, with an average of 33 respondents (95% CI 30.6–35.8).

### 2.4. Modelling Strategy

Gender was the independent variable of interest, and reference categories for analyses were non-walkers and men. Data were prepared in Stata v.14.1 [69] and analysed in *MLwiN* v.2.36 [70]. WfR was analysed as a binomial dependent variable using multilevel logistic regression through two-level random intercept Markov chain Monte Carlo (MCMC) binomial logit models (first-order marginal quasi-likelihood base estimates; burn-in = 500, chain = 50,000). First, the *average neighbourhood effects* of gender differences in WfR were estimated (adjusting for age). Second, a *random coefficient* was introduced for gender, which allowed the average association of gender and WfR to vary across neighbourhoods. Third, this model was adjusted for education, occupation, household income, residential self-selection and neighbourhood disadvantage to produce baseline estimates. Fourth, a joint Wald test was then conducted to examine whether the association between effect of gender and WfR varied significantly across neighbourhoods [70]. Fifth, *neighbourhood-level variance functions* were estimated from the fully adjusted model to examine the magnitude of between-neighbourhood variation in the probability of engaging in WfR for men and women [71]. Sixth, we incorporated a *cross-level interaction* between gender at the individual-level and each of the social environment measures at the neighbourhood-level [72], and examined reductions from the baseline model in the random coefficient for gender examined to assess whether differences in social environments explain neighbourhood differences in the association between gender and WfR [73]. Lastly, to investigate whether differences in social environments explain the between-neighbourhood variation in WfR for men and women, we incorporated each of the social environment measures to the fully adjusted baseline model as a fixed effect and assessed reductions in neighbourhood-level variance functions in the likelihood of WfR for men and for women.

## 3. Results

### 3.1. The Relationship between Gender and Recreational Walking

On average, women were more likely to walk for recreation (19% higher, 95% Credible Interval (CrI) 1.07–1.32) compared to men. 

Table 2 presents the results of the analytic steps addressing the aims of this study. Adjustment for additional covariates (including socioeconomic position, residential self-selection and neighbourhood disadvantage) attenuated the significant average neighbourhood effects of gender differences in WfR to the null (Model 1 in Table 2, OR 1.12, 95% CrI 0.99–1.27).

### 3.2. Variation of the Average Gender Differences in Recreational Walking across Neighbourhoods

The Wald test of the random coefficient for gender in the baseline model (Model 1) indicated that the relationship between gender and WfR varied significantly across neighbourhoods (*p*-value = 0.013).

### 3.3. Between-Neighbourhood Variation in the Probability of Recreational Walking for Men and Women

The neighbourhood-level variance functions in the baseline model (Model 1) revealed significant between-neighbourhood variation in WfR for both men and women, although the magnitude of the variation for women was twice that of men (0.109 and 0.050, respectively).

### 3.4. The Contribution of the Social Environment to Explaining Neighbourhood Differences in the Gender and Walking for Recreation Relationship

The cross-level interaction of gender with social cohesion (Model 3) was not statistically significant, and there was no evidence that this interaction explained neighbourhood differences in the relationship between gender and WfR. 

Likewise, the cross-level interaction of gender with incivilities (Model 5) was not statistically significant. However, significant main effects were observed for incivilities (OR 1.17, 95% CrI 1.04–1.32), although there was no evidence that this interaction explained neighbourhood differences in the relationship between gender and WfR.

The cross-level interaction of gender with perceptions of safety from crime (Model 7) was not statistically significant, and there was no evidence that this interaction explained neighbourhood differences in the relationship between gender and WfR.

### 3.5. The Contribution of the Social Environment to Explaining Between-Neighbourhood Variation in Walking for Recreation for Men and Women

Social cohesion was not statistically associated with WfR (Model 2), and its inclusion as a fixed effect accounted for none of the between-neighbourhood variation in WfR for either men or women.

Incivilities were statistically associated with WfR (Model 4; OR 1.17, 95% CrI 1.06–1.29), although their inclusion as a fixed effect had a negligible impact in explaining the between-neighbourhood variation in WfR for either men or women.

Safety from crime was not statistically associated with WfR (Model 6), and its inclusion as a fixed effect accounted for none of the between-neighbourhood variation in WfR for either men or women.

## 4. Discussion

Within the same capital city, gender differences in WfR seemed to vary significantly across neighbourhoods [32], although the reasons for this variation remain unknown. Because the social environments of neighbourhoods might influence (encourage or discourage) the recreational walking of women differently to men [23,33], this study investigated the contribution of the social environment to explaining gender differences in WfR across neighbourhoods.

As expected based on previous research [31,32,74], women were more likely than men to walk for recreation prior to adjustment for covariates (which attenuated the effects to the null). Further investigations revealed that men were more likely to be higher educated, in professional occupations and living in households with higher incomes, all of which has previously been associated with leisure-time PA and WfR [75,76]. On the other hand, women might spend more time in their neighbourhood, as they engage less in full-time employment [77] and more in caregiving and domestic activities compared to men [11,34,78], although these average neighbourhood effects could also partially reflect women’s preference for walking rather than doing vigorous PA [15]. This evidence suggests a contextual opportunity for ecological interventions that facilitate active living and reduce the gender disparity in overall PA participation through increases in WfR. 

Consistent with a previous study [32], the gender differences in WfR seemed to vary significantly across neighbourhoods, suggesting that while some neighbourhood environments might influence the WfR of men and women similarly, other environments have a differential impact. Furthermore, variation in WfR was observed between neighbourhoods for both men and women, although the magnitude of the variation for women was twice that of men. These results suggest that the neighbourhood environment might differentially shape and circumscribe the recreational walking of men and women, with women being more sensitive to environmental factors. These findings are consistent with emerging evidence across geographical settings noting stronger environmental associations in women regarding walking [79], and WfR in particular [23,74]. 

In this study, we investigated the contribution of the social environment (conceptualised through neighbourhood-level perceptions of social cohesion, incivilities and safety from crime) to explaining: (1) neighbourhood differences in the gender-WfR relationship; and (2) between-neighbourhood variation in WfR for men and women. 

Of the social environment measures considered, only higher perceptions of incivilities were significantly related to the likelihood of WfR as a main effect in our study. This unexpected finding could be due to the likely association between incivilities and other built environment features associated with more WfR. For instance, the presence of commercial land uses might attract recreational walkers as well as graffiti and rubbish. While graffiti and rubbish are often indicators of ‘fractured neighbourhoods’ and might be perceived as something to be avoided, walkers might also be attracted to view the street art. Furthermore, regular recreational walkers may be more aware of incivilities in their local environments (i.e., same-source bias) [50].

Regarding the first aim, none of the neighbourhood-level perceptions of Brisbane’s social environment produced significant cross-level interactions, nor explained neighbourhood differences in the relationship between gender and WfR. Likewise, a recent systematic review and meta-analysis of the environmental correlates of total walking did not identify any consistent moderating effects of gender [50]. However, an earlier multi-country study identified gender as a significant moderator between the perceived social environment (aesthetics and safety from crime in particular) and self-reported WfR, with women showing stronger associations than men [23]. While the evidence is inconsistent (possibly due to differences in the amount of variation in social environments across urban settings), there are indications that a more supportive social environment might be required to encourage women to walk for recreation. 

Second, the present study investigated whether—and to what extent—the neighbourhood social environment explains between-neighbourhood variation in WfR for men and women. Contrary to our hypothesis, Brisbane’s social environment did not noticeably reduce the neighbourhood-level variances in WfR for either men or women. Several studies exploring how men and women interact with perceived environments with regard to WfR have either noted inconsistent patterns [33] or have reported null findings [31,80]. However, perceived safety from crime was positively associated with total walking in a systematic review and meta-analysis [50]. Self-reported measures of the same neighbourhood can vary widely across individuals, reflecting differences in culture and walking preferences [81], which could explain the inconsistent findings across geographical settings [50].

Given the consistency of these null findings regarding the social environment measures in this investigation, we conclude that Brisbane’s social environment did not seem to contribute to the gender differences in WfR observed across neighbourhoods. There are several possible reasons for these unexpected results. As observed in earlier studies [23,82], it is likely that our findings are context-specific. Cities vary widely in their cultural and structural characteristics (such as levels of welfare support, concentration of poverty and ethnic diversity) [83], and local governments shape neighbourhood environments through planning, implementation, delivery of services, infrastructure, and policies [30]. Brisbane is a medium density urban environment characterised by low crime rates and managed by a single City Council [84], located within a high income country (Australia) with well-established welfare provisions [83]. This could explain the limited variation across Brisbane’s neighbourhoods—ranging from good to optimal—observed in the social environment measures. Furthermore, without Brisbane’s relatively safe social environment, the observed between-neighbourhood variation of gender differences in WfR might have been larger. In contrast, urban settings characterised by extreme levels of poverty, ethnic segregation and high urban crime rates like, for instance, Chicago [85], have shown to influence levels of exercise, with stronger effects seen in women [86,87]. Our results are particularly relevant within the context of the *Brisbane Vision 2031* [88], which includes the incorporation of crime prevention through environmental design practices that facilitate active, healthy communities through safe and sustainable recreational and travel choices, including walking.

Furthermore, the gender differences in WfR observed across neighbourhoods might have been better explained through social environment measures not considered in this study, such as viewing people being active, which has previously been associated with WfR [74,82], or perhaps through built—rather than social—environment features. For instance, well-maintained pedestrian infrastructure (such as sidewalks, curbs, footpaths and recreational facilities), have previously shown associations with WfR [49,89], as have aesthetics and access to public spaces [90]. Perceived residential density, land-use mix, street connectivity, and proximity to parks were linearly associated with WfR across twelve countries [49], while residential density was the only attribute associated with WfR across four urban settings in another study [23]. The quality of recreational destinations (attractiveness of, satisfaction with, or incivilities in parks and PA facilities) was consistently associated with WfR in a review [27]. Furthermore, a gender-sensitised single-level study noted that perceived built environmental factors (such as access to shops, the presence of sidewalks, and access to recreational facilities) were more important for women in regards to WfR compared to men [74].

This study has a number of limitations. While the cross-sectional design of this study limits causal conclusions, adjustment for residential self-selection (which is rare among cross-sectional neighbourhood-based studies [50,91]), ensured more reliable estimates of the influence of environmental exposures on WfR by accounting for individual-level bias (a regular recreational walker might select a residence which facilitates their WfR), and controlled—to a certain extent—for possible reverse causation [50,91,92,93]. WfR was self-reported, which is less accurate than objective measures of walking, as it might reflect recall and/or desirability bias [94]. However, objective measures lack the contextual aspects of walking, such as its purpose and location, unless combined with Global Positioning Systems (GPS) and applied algorithms [95]. Furthermore, Likert scales (used in this study to capture perceptions of the social environment) are subject to scale perception bias (reflecting demographic, interpersonal and cross-cultural preferences), which could lead to inaccurate assumptions [81]. In addition, social environment perceptions were not specifically asked in the context of WfR, and different neighbourhood boundaries vary in relevance depending on the type of PA studied (recreational vs transport walking) [50] and across demographic populations (men vs. women) [25,27]. An increased correspondence between the environmental measure, the behaviour of interest and the setting in which the behaviour takes place might produce stronger associations [96]. Finally, the limited variability in Brisbane’s social environments potentially underestimated the strength of associations between the neighbourhood social environment and WfR, limiting the generalisability of findings. However, this limited variability, along with the high average scores for the social environment measures, suggests that if the social environment is generally perceived as friendly, trustworthy, and supportive, and these perceptions are found in most neighbourhoods, this is likely to promote gender equity in walking.

Nevertheless, from an urban planning perspective, it is important to acknowledge the complexity of environmental influences on WfR. Based on previous research, women experience more individual and environmental barriers to PA participation [9,10], and the social environment appears to influence their PA more than men [11]. The marked gender disparity in PA overall participation [1,2,4] is acknowledged by WHO’s related policy frameworks [12,13] which call for ecological evidence to inform gender-responsive multilevel strategies to increase PA in populations through active living opportunities. 

## 5. Conclusions

On average, women walked more for recreation than men prior to adjustment for covariates. However, consistent with a previous investigation [32], these average associations (commonly reported in the literature) seemed to vary across neighbourhoods. In other words, neighbourhood-level factors might differentially influence the recreational walking of men and women, and women seemed more sensitive to their environments. Nonetheless, the social environment did not appear to be one of these factors in Brisbane, an urban setting where structural differences between neighbourhoods might not be as extreme as in other cities [83], hinting at other neighbourhood-level characteristics. 

This study contributes to broader debates about the important role that the neighbourhood design has in facilitating the healthy lifestyle of residents who are regularly exposed to it [97,98]. As previously advocated [99], our results favour the ongoing longitudinal multilevel analyses of demographic heterogeneity around the neighbourhood averages, as they more realistically reflect the impact of neighbourhood exposures on the walking patterns of different population subgroups. Such investigations—particularly when undertaken in urban settings characterised by larger variation in their social and built environments—can inform ecological interventions which facilitate WfR opportunities everywhere for both men and women, resulting in sustainable public health, socioeconomic and environmental gains for the overall population [100]. Such neighbourhood-level interventions can ultimately support the achievement of WHO’s objective of a global 10% reduction in the prevalence of physical inactivity by 2025 [12] as well as the Sustainable Development Goals [101], particularly Gender Equality (Goal 5) and Sustainable Cities and Communities (Goal 11). 

## Figures and Tables

**Table 1:** Sociodemographic characteristics of the analytic sample by gender and minutes of recreation walked: 2009 HABITAT data.

Sociodemographic Characteristics	Men			Women		
	Total	0 mins	1–840 mins	Total	0 mins	1–840 mins
Total (*n*)	2844	859	1985	3799	1011	2788
	*n*	%	%	*n*	%	%
**Age**						
42–50 years	1152	30.8	69.2	1349	26.9	73.1
51–59 years	997	31.2	68.8	1416	26.6	73.4
60–67 years	695	27.8	72.2	1034	26.3	73.7
**Education**						
Bachelor’s degree or higher	988	24.6	75.4	1203	23.1	76.9
Diploma/associate degree	340	25.0	75.0	421	21.4	78.6
Certificate	620	34.2	65.8	548	24.6	75.4
No post-school qualification	896	35.6	64.4	1627	31.2	68.8
**Occupation**						
Professional	1071	26.3	73.7	1077	22.7	77.3
White collar	336	25.6	74.4	980	28.0	72.0
Blue collar	630	42.2	57.8	204	35.8	64.2
Not in workforce	506	26.1	73.9	1055	25.4	74.6
Not easily classifiable	301	30.9	69.1	483	31.5	68.5
**Income**						
$130,000+	664	23.8	76.2	580	23.3	76.7
$72,800–129,999	811	27.4	72.6	889	26.5	73.5
$52,000–72,799	395	35.2	64.8	519	27.4	72.6
$26,000–51,999	465	32.9	67.1	702	25.6	74.4
Less than $25,999	234	34.2	65.8	478	34.1	65.9
Not classified	275	38.9	61.1	631	24.6	75.4

**Table 2:** Gender differences in recreational walking, variation of this relationship across neighbourhoods, and the contribution of the social environment to explaining this variation.

Effects	Baseline		Perception of Social Cohesion				Perception of Incivilities				Perception of Safety from Crime			
	M1		M2		M3		M4		M5		M6		M7	
*Fixed Effects ^a^*	OR	95% CrI	OR	95% CrI	OR	95% CrI	OR	95% CrI	OR	95% CrI	OR	95% CrI	OR	95% CrI
Men	1.00	--	1.00	--	1.00	--	1.00	--	1.00	--	1.00	--	1.00	--
Women	1.12	0.99, 1.27	1.12	0.99, 1.28	1.12	0.98, 1.27	1.12	0.99, 1.28	1.12	0.98, 1.28	1.12	0.98, 1.27	1.12	0.98, 1.27
L2 exposure ^b^	--	--	0.99	0.91, 1.07	0.96	0.87, 1.06	**1.17**	**1.06, 1.29**	**1.17**	**1.04, 1.32**	0.91	0.83, 1.01	0.91	0.81, 1.03
*Interactions*														
Males	--	--	--	--	1	--	--	--	1	--	--	--	1	--
L2 * women ^c^	--	--	--	--	1.07	0.94, 1.21	--	--	1.00	0.87, 1.14	--	--	1.00	0.88, 1.14
*Random effects*														
Random coefficients (s.e.) ^d^														
Men	--		--		--		--		--		--		--	
Women	**0.080 (0.036)**		**0.083 (0.038)**		**0.081 (0.036)**		**0.083 (0.036)**		**0.081 (0.036)**		**0.084 (0.036)**		**0.082 (0.036)**	
*p*-value	**0.013**		**0.014**		**0.012**		**0.011**		**0.013**		**0.011**		**0.016**	
Variance functions (s.e.) ^e^														
Men	**0.050 (0.018)**		**0.051 (0.019)**		**0.050 (0.018)**		**0.049 (0.017)**		**0.049 (0.018)**		**0.051 (0.019)**		**0.051 (0.018)**	
Women	**0.109 (0.025)**		**0.110 (0.025)**		**0.110 (0.025)**		**0.107 (0.024)**		**0.107 (0.025)**		**0.109 (0.025)**		**0.109 (0.025)**	

Note: Boldface indicates significance. CrI: Credible Interval. Model 1: Gender differences in the likelihood of WfR (randomised at the neighbourhood level), adjusted for age, socioeconomic position (education, occupation and household income), residential self-selection and neighbourhood disadvantage. Models 2, 4 and 6: M1 + each of the social environment measures entered into the models separately. Models 3, 5 and 7: M2, M4 and M6 + cross-level interactions of gender with each of the social environment measures. ^a^ Fixed effects capturing the neighbourhood average (pooled) effects of gender differences in the likelihood of WfR. ^b^ L2 exposure: Main effects for each level 2 environmental exposure, i.e., social cohesion in M2 and M3, incivilities in M4 and M5, and safety from crime in M6 and M7. ^c^ The * indicates an interaction between the level 2 predictor and women. ^d^ Random coefficient (with standard error) testing whether the gender differences in the likelihood of WfR are the same everywhere (reflecting the average effect) or whether the relationships vary across neighbourhoods (thus, the neighbourhood-level variance functions are reported in grey). ^e^ Variance functions capturing the extent of between-neighbourhood variation in WfR for males and females (thus, the random coefficients are reported in grey).
